# Study protocol of a mixed-methods evaluation of a cluster randomized trial to improve the safety of NSAID and antiplatelet prescribing: data-driven quality improvement in primary care

**DOI:** 10.1186/1745-6215-13-154

**Published:** 2012-08-28

**Authors:** Aileen Grant, Tobias Dreischulte, Shaun Treweek, Bruce Guthrie

**Affiliations:** 1 Quality, Safety and Informatics Research Group, Population Health Sciences, University of Dundee, Mackenzie Building, Dundee, DD2 4BF, UK; 2 Medicines Management Unit, NHS Tayside, c/o University of Dundee, Mackenzie Building, Dundee, DD2 4BF, UK

**Keywords:** Complex intervention, Process evaluation, Protocol, Mixed methods, Randomized controlled trial

## Abstract

**Background:**

Trials of complex interventions are criticized for being ‘black box’, so the UK Medical Research Council recommends carrying out a process evaluation to explain the trial findings. We believe it is good practice to pre-specify and publish process evaluation protocols to set standards and minimize bias. Unlike protocols for trials, little guidance or standards exist for the reporting of process evaluations. This paper presents the mixed-method process evaluation protocol of a cluster randomized trial, drawing on a framework designed by the authors.

**Methods/design:**

This mixed-method evaluation is based on four research questions and maps data collection to a logic model of how the data-driven quality improvement in primary care (DQIP) intervention is expected to work. Data collection will be predominately by qualitative case studies in eight to ten of the trial practices, focus groups with patients affected by the intervention and quantitative analysis of routine practice data, trial outcome and questionnaire data and data from the DQIP intervention.

**Discussion:**

We believe that pre-specifying the intentions of a process evaluation can help to minimize bias arising from potentially misleading post-hoc analysis. We recognize it is also important to retain flexibility to examine the unexpected and the unintended. From that perspective, a mixed-methods evaluation allows the combination of exploratory and flexible qualitative work, and more pre-specified quantitative analysis, with each method contributing to the design, implementation and interpretation of the other.

As well as strengthening the study the authors hope to stimulate discussion among their academic colleagues about publishing protocols for evaluations of randomized trials of complex interventions.

**Data-driven quality improvement in primary care trial registration:**

ClinicalTrials.gov: NCT01425502

## Background

Trials of complex interventions are often criticized as being a black box because it is difficult to know exactly why an intervention did (or did not) work. The UK Medical Research Council’s framework for designing and evaluating complex interventions recommends conducting a process evaluation in order to ‘explain discrepancies between expected and observed outcomes, to understand how context influences outcomes, and to provide insights to aid implementation [1].’ However, when designing our own process evaluation of a cluster randomized trial of a complex intervention, [2] we found little specific guidance. In response, we have proposed a framework to guide process evaluation design [3].

Pre-specifying and publishing protocols are recognized as improving the standards of clinical trials, by allowing comparison of what was intended with what was actually done. Although process evaluations are somewhat different (since flexibility to evaluate unexpected findings qualitatively will often be useful), we believe that publication of process evaluation protocols is also best practice, and that pre-specifying the intentions of process evaluations can help to minimize bias arising from potentially misleading post-hoc analysis. Unlike trial protocols, there is little explicit guidance or standards for the reporting of process evaluation protocols. We hope publishing this protocol will help stimulate discussion among the academic community.

This paper briefly describes the trial being evaluated, presents a logic model detailing the trial hypothesized pathway of change, and states the research questions that arise from our assumed model of trial processes, overall study design, and detailed methods for answering each question.

### Data-driven quality improvement in primary care trial

High-risk prescribing of non-steroidal anti-inflammatory drugs (NSAIDs) and antiplatelet agents account for a significant proportion of hospital admissions, due to preventable adverse drug events [4,5]. We are conducting a randomized controlled trial of a complex intervention to improve prescribing safety of these drugs in 40 general practices in two Scottish Health Boards (the DQIP trial). The trial is fully described in the published protocol [2]. In brief, the DQIP intervention comprises three components. The first component is a web-based informatics tool that provides weekly updated feedback of targeted prescribing at practice level. This prompts the review of individual patients affected and summarizes each patient’s relevant risk factors and prescribing. The second component is an educational outreach visit that highlights the risks of the targeted prescribing and provides training in the use of the tool. The final component is a fixed payment of 350 GBP (560 USD; 403 EUR) to each practice up front and a fee-for-service payment of 15 GBP (24 USD; 17 EUR) for each patient reviewed.

The trial has a stepped wedge design, [6,7] where all participating practices receive the DQIP intervention. These practices are randomized to one of ten different start dates, with each practice functioning as its own control in a time series analysis. The primary outcome measure is a composite of nine measures of high-risk NSAID and antiplatelet prescribing. A separate economic evaluation is planned.

### Structure/framework of DQIP parallel process evaluation protocol

**Figure 1:**
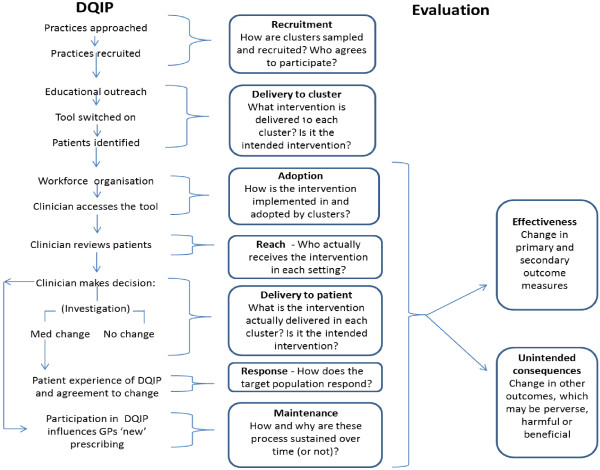
**Data-driven quality improvement in primary care** (**DQIP) trial hypothesized pathway of change and process evaluation model.**

Drawing on our proposed framework for designing process evaluations of cluster randomized trials, [3] Figure 1 shows the processes we assume have to occur for the intervention to be effective, and therefore explicitly identifies targets for evaluation. This logic model underlies the process evaluation design and data collection, although resource constraints inevitably require prioritization.

### DQIP process evaluation aim and research questions

#### Aim

The aim of this study is to understand DQIP delivery, implementation and translation into reducing high risk prescribing.

Research questions:

1. How do different practices adopt the intervention and how does this influence their delivery to targeted patients and whether they continue to use the intervention? Are there any positive or negative unintended consequences from a practice perspective?

2. How do patients respond to the intervention? Are there any positive or negative unintended consequences from a patient perspective?

3. How are practice organizational characteristics associated with variation in recruitment, adoption, reach, delivery to the patient, and maintenance?

4. Does effectiveness vary between practices, and if so, how are practice characteristics and processes including structure, adoption, reach, delivery to the patient, and maintenance associated with effectiveness?

### Management and governance

Research ethical approval for this study was granted by Fife and Forth Valley Research Ethics Committee (11/AL/0251). An external Trial Steering Committee and an overall Program Advisory Group, consisting of independent professional and lay people, have been established and both meet bi-annually.

## Methods/design

### Overall study design

The study is a mixed-methods parallel process evaluation. It will include a qualitative dominant case-study approach in a purposeful sample of practices and a hypothesis-testing quantitative analysis of data from all practices. Data collection and analysis will be conducted in parallel to the DQIP trial itself.

### Methods for research question 1

#### Study design

Study will be a primarily qualitative, mixed-methods in-depth case-study in a sample of eight to ten practices.

### Sampling

Sampling for this study will be purposive and ensure heterogeneity in list size, reflecting our prior expectation that effectiveness will be greater in small practices; [8] and initial use of the tool (since this is how patients for review are identified and so a key process if our logic model is true); and the date the practice started the trial (which is randomized, but workload in general practice is strongly seasonal, so resource to deliver the intervention may vary by start date). We will sample two practices from the first two cohorts of starting date and develop theories that can then be tested in the subsequent case studies [9].

### Qualitative data collection

Data collection will use a variety of methods, in order to understand how intervention processes were perceived and implemented in each practice, and how these processes influence observed trial outcomes [10]. As part of the intervention in all practices, the qualitative researcher will assist the research pharmacist in delivering the education and training to the trial practices, and make field notes detailing attendance and the practice’s response. Any additional communication or practice visits will be recorded in the same way. These data will inform the focus of the interviews in each practice, and additionally be used to help construct a detailed, ‘rich’ description of each practice for cross-case analysis [11].

General practitioners (GPs), practice pharmacists and practice managers, from the case-study practices, involved in delivering the DQIP intervention to patients will be invited to interview approximately six months after the practice starts the trial. Depending on practice size and how each practice has organized DQIP work, we will interview one to three professionals per practice. At a minimum, the GP most involved in DQIP will be interviewed, but other GPs involved, the practice manager and the practice pharmacist will also be interviewed. Participants will be asked about their experience of the intervention, the practice process for review, how the intervention fitted with their existing work, barriers and facilitators, maintenance over time, and any perceived unintended consequences. Where possible, the GP most involved in DQIP will be re-interviewed three to six months later, to explore changes over time and the factors involved in maintaining and sustaining use of the tool. Interviews will be conducted at a time and place of the participant’s choosing, facilitated by a topic guide drawing on the logic model and Normalization Process Theory (NPT), [12] and with the participant’s permission, audio-recorded for transcribing. Normalization Process Theory is a sociological theory designed to understand how practices or interventions are implemented and become embedded into everyday work (normalized and routinized). NPT is made up of four domains, coherence, cognitive participation, collective action, reflective monitoring, with each of these domains divided into four categories [12].

### Qualitative data analysis

The professional qualitative data will be inductively and deductively analyzed in separate NVivo 8 (QSR International, Southport, UK) databases. Deductive analysis will be by examining the data through the lens of NPT [12]. Our pilot work has illustrated that this means of analysis sometimes ‘divides’ emergent codes in the data. As a result, the data will be analyzed inductively again, to allow themes to emerge from the data and to explore issues that the NPT deductive analysis may be less suited to identify and examine.

### Case-study analysis

For each practice (case), we will have a set of quantitative and qualitative data which can be synthesized. Quantitative data, as described in detail below, will be used to descriptively characterize each practice in terms of *practice structural characteristics,* and measures of *adoption, delivery to patient, reach, and maintenance*. Qualitative data includes field notes relating to research team contacts with each practice and interview data. Synthesis of the qualitative and quantitative data for the case studies will take two forms; first a comprehensive, ‘rich’ description of each case-study practice will be created based on all data, then this description will be used to conduct a cross-case comparison based on the logic model [13]. The aim of this analysis is to examine in detail how practices implement (or not) the processes that our logic model assumes are required to deliver change in high-risk prescribing, and how these processes appear to influence effectiveness in each practice. The aim of this sequential merging of qualitative and quantitative data is not triangulation but crystallization where we are using the qualitative data to explain the quantitative [14,15]. This offers the advantage of allowing the qualitative data to explore complex, unexpected or contradictory quantitative data collected from the tool and adoption questionnaires. A constant comparison and deviant case approach will be adopted [16]. Constant comparison will allow the researcher to test the emerging hypothesis by comparing different case-study practices. Any deviant examples from the emergent hypothesis will be examined. Analysis will initially be simultaneous with data collection to allow the emerging analysis to be tested and revised in subsequent data sampling, collection and analysis. The framework [17] analysis technique will be used to support within-case and cross-case analysis.

### Methods for research question 2

#### Study design

The design is that of a focus group study involving a sample of patients targeted by the intervention.

### Sampling

Four to six of the case-study practices will invite patients who have been targeted by the intervention to attend a focus group.

### Data collection

One focus group per practice will be held. The focus group will have two stages. First, the patients will be asked about their experience of medication reviews and the extent to which they were aware of the intervention (*response*). Then after watching a short presentation about the way clinical data is being used in this study, patients will be asked about their experience, perceptions and any consequences of the intervention triggered by review or medication change and their attitudes and beliefs about the way in which clinical data is being used.

### Data analysis

Patient focus group data will be analyzed inductively to allow themes to emerge from the data. The framework technique [17] will be used to facilitate analysis.

### Methods for research questions 3 and 4

#### Study design

The study requires quantitative analysis of routinely available data about practices, trial process data generated by DQIP informatics tool, practice questionnaire data, and trial outcome data. We hypothesize that practice *organizational characteristics* and *adoption* will be associated with the level of *reach, delivery to the patient* and *maintenance* achieved, and that lower levels of *reach*, *delivery to the patient* and *maintenance* will be associated with lower *effectiveness*. The specific hypotheses to be tested will be based on findings from the case studies.

### Sampling

Data will be collected for all practices.

### Data collection

Multiple datasets will be collected and linked. *Practice characteristics* will be measured using routinely available data including list size, rurality , deprivation, proportion of older patients, postgraduate training status, dispensing status, contract type (nGMS, section 17c/2c), Health Board, Community Health Partnership, overall QOF clinical performance, and QOF performance on relevant medicines management indicators (Medicines 6, 10, 11, 12){National Institute of Clinical Excellence, 2012}. Baseline levels of the high risk NSAID and antiplatelet prescribing being targeted will also be available for participating practices. During the *recruitment of clusters* we will document the trial recruitment process and carefully record those practices that are approached, those that respond and those that are recruited. Participating and non-participating practices will be compared using available *practice characteristic* data. *Adoption* will be measured using a quantified assessment of engagement with the education outreach based on which practice members attended and field notes of the interaction, and by a survey instrument developed for the trial and completed at trial start and after six to nine months based on NPT. [12] The adoption questionnaire is based on the four domains of NPT, with the baseline survey focusing on coherence and cognitive participation, and the follow-up survey collecting additional data on collective action and reflective monitoring. *Reach* will be measured as the proportion of patients identified by the informatics tool who are actually reviewed. *Delivery to the patient* will be measured in terms of patterns of review recorded in the tool (which measures were focused on, how they were carried out [records review, face-to-face consultation, telephone consultation]), the decisions made (the proportion of patients who decide to continue, the proportion who decide to continue with gastric protection and the proportion who decide to stop the drug). *Maintenance* will be measured in terms of how reach changes over time. *Effectiveness* will be measured in terms of the trial primary outcome (the high risk NSAID/antiplatelet composite) and the two secondary outcomes of ‘repeated’ and ‘new’ prescribing (since the intervention targets the review of repeated prescribing, with an expectation that the experience of reviewing will reduce new prescribing as well, but that this may vary by practice and particularly by practice size).

### Data analysis

Initial descriptive analysis will use *practice characteristics* data to compare participating practices with the wider population of practices in the two Boards and nationally (to assess the representativeness of *recruited* practices and the implications for generalizability). The overall extent of *reach*, *delivery to the patient* and *maintenance* will then be examined, and univariate and multivariate associations with *practice characteristics* and *adoption* will be examined using cross-tabulations, comparison of means, and logistic/linear regression as appropriate to the data. The extent of variation between practices in the three specified measures of *effectiveness* will be examined using multilevel logistic regression, and associations between *effectiveness* and *practice characteristics*, *adoption*, *reach*, *delivery to the patient*, and *maintenance* will be examined.

### Synthesizing results from all three studies

Synthesis across the three studies will have several elements:

1. The quantitative study will inform case-study sampling and will help contextualize sampled settings.

2. The case and patient focus group studies will provide a rich understanding of how the intervention was perceived and implemented, and how actual implementation related to our model of how the intervention was intended to work. These data will generate hypotheses for testing in the quantitative study, and will provide the flexibility either to identify key processes on the assumed pathway quantitatively or to identify unexpected processes that are not in the logic model, or both.

3. The quantitative study will test hypotheses generated from the qualitative analysis, and help inform judgments about generalization of the case and focus group studies.

4. Overall synthesis will draw on findings from all three studies, which will be used to construct a narrative synthesis of the process of implementation of the intervention, to contextualize the main trial findings (whether positive or negative) and to inform judgments about external validity and generalizability , real-world implementation if appropriate, and further intervention development [18].

## Discussion

We have piloted these data collection methods in four general practices and in six focus groups with patients from these practices and with members of the public. These methods have been acceptable to practitioners and patients and have generated sufficient data to feasibly evaluate this complex intervention trial. The pilot has influenced the focus and design of this evaluation and the associated topic guides and questionnaires.

As in any study, choices have been made in the design of this process evaluation protocol that balance ideal research questions with feasibility and resource constraints. [19] We believe that a prior design of the process evaluation that maps to expectations about how the intervention will work strengthens the study. It is also important, however, to retain the flexibility needed for examining the unexpected and the unintended. From that perspective, a mixed-methods evaluation allows the combination of exploratory and flexible qualitative work, and more pre-specified quantitative analysis, with each method contributing to the design, implementation and interpretation of the other [20]. Design is always constrained by the resources available though, and although we have been fortunate in being relatively well-resourced for this element of the project, our chosen case-study method does mean having to sample a relatively small number of practices to carry out the qualitative work. Inevitably though, extending the interviewing to a larger number of practices would involve less depth in each practice, and we chose to prioritize more depth in a case-study approach using multiple interviews in selected practices.

We have also chosen to conduct a parallel process evaluation, where data collection is simultaneous with trial implementation [21]. A key advantage is that data collection is contemporaneous with the practices actually doing the work of implementation, but a potential disadvantage is the risk that the case-study practices vary little in their overall effectiveness in delivering main trial outcomes. A post-hoc design that samples based on main trial outcomes would avoid this risk, but at the cost of being designed after trial effectiveness is known, and conducted long after the trial completes. Overall, we judged a parallel process evaluation to be more likely to produce robust findings, and although the protocol is pre-specified, the methods proposed are flexible to unexpected findings in that qualitative data collection and analysis is iterative in nature, and will inform the choice of the actual hypotheses to be tested quantitatively. A final potential weakness is that some of the quantitative data is relatively thin. For example, the data on the nature of review carried out is collected routinely in the DQIP informatics tool but lacks contextual information or much detail. This reflects a trade-off between being able to collect data in all practices and avoiding burdening practices with data collection that has no clinical purpose.

Our study offers the opportunity to explain why or why not the DQIP intervention reduced high risk prescribing. If the intervention does not reduce high-risk prescribing as intended, then the analysis will help identify why this happened in terms of where the assumptions of the logic model broke down, or call into question the underlying model. If the intervention does reduce high-risk prescribing, then the process evaluation will provide information to inform judgments about likely generalizability, and identify if improvement was general or restricted to some practices which may influence real-world implementation or identify targets for a modified intervention.

## Trial Status

Active. Trial started 31st October 2011.

## Abbreviations

DQIP: data-driven quality improvement in primary care; GP: general practitioner; NPT: Normalization Process Theory; NSAIDS: non-steroidal anti-inflammatory drugs; QOF: Quality and Outcomes Framework.

## Competing interests

The author(s) declare that they have no competing interests.

## Authors’ contributions

AG and BG were involved in the initial conceptualization and study design. AG and BG developed the data collection methods; AG piloted these with the help of TD. AG and BG wrote the paper. All authors read and approved the final manuscript. AG is responsible for this manuscript.
